# Diagnosing cancer earlier: reviewing the evidence for improving cancer survival: The National Awareness and Early Diagnosis Initiative in England: assessing the evidence 5 years on

**DOI:** 10.1038/bjc.2015.23

**Published:** 2015-03-03

**Authors:** S C Hiom

**Affiliations:** 1 Cancer Research UK, Angel Building, 407 St John Street, London EC1V 4AD, UK

Since publication of the BJC supplement, volume 101; ‘Evidence for a National Awareness and Early Diagnosis Initiative' (NAEDI) in December 2009, there has been considerable investment and significant research, data collection, analysis, policy activity and application of interventions under the auspices of NAEDI. We have come a long way in our understanding of the role early cancer diagnosis has in improving survival; we have deeper insights as to what might be done to achieve it and better ways to measure progress. The 2009 supplement presented diverse evidence relevant to early diagnosis, linking late diagnosis with poor survival and ‘avoidable deaths', going so far as to quantify this (Abdel-Rahman *et al*, 2009) – figures that have been used to drive increased spending and activity in the area since. Five years on, we present selected primary research, reviews, evaluations and discussion pieces assessing the recent evidence available for earlier diagnosis and suggesting areas for further research. This introduction aims to bring the reader up to date, offers a contemporary view of our efforts to guide action to improve survival and reduce premature mortality from cancer and notes key findings from papers within this supplement (BJC, 2015).

NAEDI was launched in November 2008 (Richards, 2009), and it remains to be co-chaired by the National Cancer Director and Cancer Research UK's Chief Executive, with close involvement from the Department of Health and a wide variety of partners across the health and third sectors, as well as a burgeoning research community.

Its main aim is to address poor cancer survival by reducing the number and proportion of cancers diagnosed and treated at a late stage, mainly concentrating on symptomatic presentation and improvements across the diagnostic pathway. This is not to say that screening programmes, the development of new technologies or biomarkers, are not a vital part of the early diagnosis armoury, but simply that these are dealt with elsewhere and NAEDI concerns itself with sharing best practice and applying new intelligence to optimising pathways, approaches and behaviours. Our knowledge of the cancer types for which symptoms are indicative of ‘early'-stage disease (as opposed to when the disease is already advanced) is far from complete and remains an important focus for future research if we are to apply efforts for greatest effect where they will most likely improve cancer outcomes. The 2007 Cancer Reform Strategy (Department of Health, 2007) and subsequent Improving Outcomes Strategy for Cancer (Department of Health, 2011) placed a deliberate focus on the role of primary care in diagnosing cancer earlier, and research and understanding in this area have grown apace.

The National Health Service (NHS) reforms of 2012 saw the end of a number of bodies and functions with responsibility for taking forward the NAEDI agenda, as well as creation of NHS England and Public Health England, both of which are essential partners in our efforts within England to detect, diagnose and treat cancers as swiftly and effectively as possible. One such partnership in England has been the development and execution of ‘*Be Clear on Cancer'*, a series of public awareness campaigns whose aim, along with other related activities, is to increase public knowledge of key cancer signs and symptoms in order to encourage swifter presentation to primary care and thus more timely investigation and diagnosis. An extensive evaluation of the national lung cancer campaign was recently published in this journal (Ironmonger *et al*, 2015), whereas two further assessments of the first national bowel and lung cancer campaigns are presented in this supplement. The first (Moffat *et al*, 2015) looks at the impact on socio-demographic inequalities in awareness and GP attendance, and the second assesses the change in knowledge and perceived barriers to help-seeking (Power and Wardle, 2015). In Scotland, *‘Detect Cancer Earlier'* has been implemented with similar objectives (The Scottish Government, 2014) and Cancer Research UK, among others, is working with health departments in Wales and Northern Ireland on future possibilities.

Since the 2009 BJC supplement, research into early diagnosis has increased in both volume and scope, with new funding streams created with the express purpose of facilitating creation of a body of evidence to underpin activity to address late diagnosis and a consolidated research community. Being a complex and truly multidisciplinary research area, it combines behavioural science, primary-care research, epidemiology, policy and health services research, international comparison studies, data analysis and practice evaluation. This is crucial when you consider the original ‘NAEDI hypothesis' (Richards, 2009) updated here, indicating the multifactorial and often nonlinear nature of the pathways to diagnosis from the first onset of symptoms and the individual's response, to help-seeking, health professional interaction, to onward referral, diagnosis and beyond (Figure 1).

Opportunities for ‘delay' can occur at any or all of the points along these pathways (Walter *et al*, 2012; Weller *et al*, 2012). A study of patients with symptoms suspicious of lung cancer presented here (Walter *et al*, 2015) is the first of its kind in using a large prospective cohort and identifying factors that prompt earlier action, from the individual or the health professional, before diagnosis. It illustrates well the challenging nature of nonspecific symptoms and how to most effectively deal with them. Inequalities and variations in the time between noticing symptoms and contacting a doctor, the ‘appraisal interval', demands an understanding of what underpins them, and the review of symptom appraisal models in this supplement (Whitaker *et al*, 2015) aims to help explain and guide future research and interventions. We then present a new analysis of the relative lengths of the patient and primary-care intervals in patients with 28 common and rarer cancers (Lyratzopoulos *et al*, 2015a), showing great variation between cancer types and offering further insight into how interventions could be targeted.

A number of significant advances, made since publication of the original supplement, have enabled more accurate and timely assessment of our progress in early diagnosis, improved our understanding and also underpinned extensive further studies and analyses, examples of which are shared here. Arguably the most critical of these, by the English National Cancer Registration Service (NCRS), has been the improvement of our national staging data, the quality, completeness and timeliness of which is now reliable enough to produce national quality indicators as used in public health (Public Health England, 2015) and the health service (NHS England, 2014). This allows us to assess reduction of late-stage diagnosis, the ultimate outcome if we are to improve cancer survival and mortality through our early diagnosis efforts. Next, the NAEDI hypothesis prompted Cancer Research UK to ask the question ‘through which routes are patients diagnosed with cancer if they are not one of the 5–10% diagnosed via screening programmes?' This led to work with the National Cancer Intelligence Network (NCIN) linking registry data with Hospital Episode Statistics that became the often-cited *‘Routes to Diagnosis'* study (Elliss-Brookes *et al*, 2012; McPhail *et al*, 2013). This indicated that for cancer patients diagnosed between 2006 and 2010 a significant proportion of the patients overall are diagnosed via an ‘emergency' route, an unexpected and somewhat concerning finding that has created significant policy interest and subsequent activity to reduce emergency presentations (NHS England, 2014). The study also showed that just under a quarter were diagnosed by the 2-week wait (2WW) and a similar proportion through ‘routine' GP referrals. A subsequent in-depth analysis of the ‘Cancer Waits' database presented the relationship between referral, conversion and detection rates (Meechan *et al*, 2012), and suggested that a ‘quality measure' exists where GPs are detecting a high proportion of cancers via a 2WW *and* a large proportion of their 2WW referrals convert to cancer cases. Work is underway to explore how these relate to patient outcomes, and we are challenged to identify what distinguishes the ‘better performing' practices. The first national audit of cancer diagnosis in primary care (Rubin *et al*, 2011) was undertaken in 2009-10, the results from which were used to inform commissioning, redesign services locally and underpin continuous professional development for GPs. We present here just a small selection of the recent relevant primary-care research.

A qualitative study of 55 GPs and thematic analysis (Green *et al*, 2015) and a synthesis of significant event audits to examine the reasons for emergency presentation (Mitchell *et al*, 2015) offer insights into cancer diagnosis in primary care and possible opportunities for intervention. A rigorous assessment of an English national initiative for early diagnosis in primary care is also reported (Rubin *et al*, 2015), showing a positive impact on urgent referral rates and the critical role of clinical leadership in such initiatives. The use of clinical support tools for more prompt and accurate referral of potential cancer symptoms was discussed in the 2009 supplement (Hamilton, 2009), and it continues to be a popular line of enquiry. We report a feasibility study of the use of IT for the identification of suspected colorectal cancer in primary care (Kidney *et al*, 2015), suggesting that searching of electronic patient records is feasible. An exploratory study using simulated consultations with Australian GPs (Chiang *et al*, 2015) goes on to examine what the potential barriers might be to implementation of a ‘QCancer' risk tool in practice.

Before moving into the final section of this supplement that offers a variety of reviews and studies quantifying various elements of early diagnosis activity, we present a perspectives article that aims to understand how missed opportunities for timely cancer diagnosis occur and provide a theoretical basis for the development of future interventions (Lyratzopoulos *et al*, 2015b). This is followed by a systematic review of the literature on the association between diagnostic intervals and cancer outcomes (Neal *et al*, 2015), reinforcing that there is variation between cancer types and that considerably more quality research is needed.

Another major undertaking launched since the original BJC supplement, under the auspices of NAEDI, is the International Cancer Benchmarking Partnership (ICBP; Butler *et al*, 2013, Cancer Research UK, 2014) funded initially by the English Department of Health and set up between UK nations and others globally with population-based cancer registration and comparable health systems. A seminal publication from this partnership (Coleman *et al*, 2011) clarified that, although cancer survival has been improving in all ICBP member jurisdictions, for people diagnosed with lung, colorectal or ovarian cancer in the United Kingdom between 1995 and 2007 the survival gap did not close. For breast cancer, results indicated that we were approaching the survival of the best, with the gap closing over this period. Across the board, our survival is worse in the first 12 months following diagnosis and for patients aged 65 years and older. The ICBP goes further than measuring the survival differences, having been set up to explore the possible factors that are responsible for these differences, be they public awareness, attitudes and beliefs (Forbes *et al*, 2013), primary care (Rose *et al*, submitted) or stage-related differences (Maringe *et al*, 2012, 2013; Walters *et al*, 2013a, 2013b). Essentially, the stage comparisons indicate that for lung and colorectal cancer UK patients are diagnosed at a later stage than those in the ‘better performing' partner countries, whereas for breast and ovarian cancers UK stage distribution is not significantly different from those countries with better survival. Since both colorectal and lung cancers are very common and cancers for which our survival ‘deficit' is pronounced, this study reinforced the issue of late diagnosis in the UK. However, specifically for breast and ovarian cancers, stage for stage UK patients also do worse and this is especially true for the most advanced cancers and our older patients. With our aging population being the main cause of the lifetime risk of cancer in Great Britain now rising to one in two (Ahmad *et al*, 2015), planning and managing cancer care for older patients is crucial.

An examination of stage at diagnosis and early mortality using national registry data for patients with breast, colorectal, lung, prostate and ovarian cancers (McPhail *et al*, 2015) shows a different stage pattern for breast, prostate and colorectal cancers than for lung and ovarian cancers, and supports the finding that efforts should be concentrated on minimising stage 4 diagnosis of any cancer, as well as better understanding and reducing variation in stage-specific survival overall. Two further studies use East of England stage data linked to other data sources for patients diagnosed between 2006 and 2010 to quantify the potential survival gains of reducing socioeconomic and sex inequalities in stage for melanoma (Rutherford *et al*, 2015b) and age inequalities in stage for older breast cancer patients (Rutherford *et al*, 2015a). The supplement concludes with a study of the cancer-specific variation in emergency presentation by sex, age and deprivation across 27 common and rarer cancers (Abel *et al*, 2015). All these, and similar, studies are indicative of the types of valuable quantification possible, given the ability to link related data sets, something that has become increasingly difficult to achieve of late.

In conclusion, our understanding of what underpins poor cancer survival and premature mortality has grown considerably since publication of the original NAEDI supplement, but there remain gaps in our knowledge that demand further research. The ‘size of the prize' for early diagnosis was previously estimated as a proportion of the 10 000 avoidable deaths annually, based on survival comparisons between GB and the ‘best performing' European countries (Abdel-Rahman *et al*, 2009). Although we do not know the precise proportion affected by earlier diagnosis – as within-stage variation implies that treatment differences also play a part – it is clear that optimal and ‘curative' treatment can only be offered to patients diagnosed at an early enough stage to benefit from it. Furthermore, as incidence of cancer continues to rise with our ageing population, so too will numbers represented by any given survival gap; however, even this gap does not account for all the gains to be made, as no country has ‘perfect' cancer survival. We can therefore conclude that pursuit of earlier diagnosis and optimal treatment can lead to many thousands of patients across the United Kingdom being spared the trauma of a late diagnosis and their life being cut short by cancer. It is also clear from national variation and inequalities that significant improvements can be made just by sharing best practice and using the knowledge, interventions, tools and clinical expertise available today, even before the realisation of promising new molecular techniques, innovations and personalised medicines. At the time of submission of this 2015 supplement, a new Cancer Taskforce has been announced in England, chaired by the Chief Executive of Cancer Research UK. Evidence and understanding generated through NAEDI will undoubtedly help underpin development of its new cancer strategy.

## Figures and Tables

**Figure 1:**
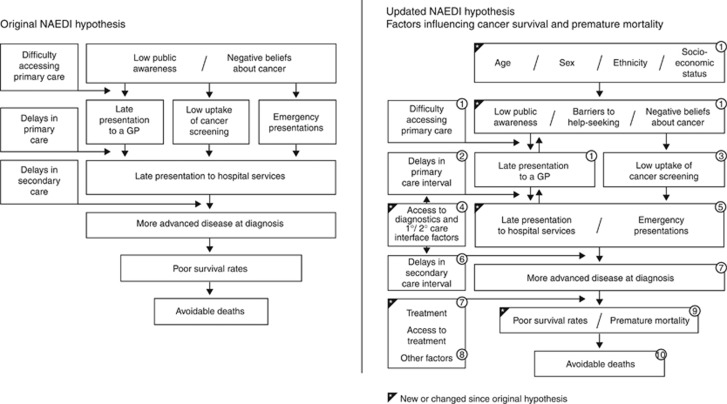
**Numerous references exist supporting the updated NAEDI hypothesis, some of which are published in this supplement.** Other relevant references include: (1) Robb *et al*, 2009; Waller *et al*, 2009; Quaife *et al*, 2014; Keeble *et al*, 2014; (2) Lyratzopoulos *et al*, 2012, 2013; (3) Von Wagner *et al*, 2011; Lo *et al*, 2014; (4) Shawihdi *et al*, 2014; (5) Elliss-Brookes *et al*, 2012; Mitchell *et al*, personal communication; (6) Cancer Waiting Times; NHS England, 2015; (7) Maringe *et al*, 2012, 2013; Walters *et al*, 2013a, 2013b; National Cancer Intelligence Network (2015); (8) McPhail *et al*, 2013; (9) De Angelis *et al*, 2014; Allemani *et al*, 2014; Coleman *et al*, 2011; and (10) Abdel-Rahman *et al*, 2009.
